# Ordinal Patterns in Heartbeat Time Series: An Approach Using Multiscale Analysis

**DOI:** 10.3390/e21060583

**Published:** 2019-06-12

**Authors:** María Muñoz-Guillermo

**Affiliations:** Departamento de Matemática Aplicada y Estadística, Universidad Politécnica de Cartagena, 30202 Cartagena, Spain; maria.mg@upct.es

**Keywords:** ordinal patterns, multiscale analysis, heartbeat time series, rényi entropy

## Abstract

In this paper, we simultaneously use two different scales in the analysis of ordinal patterns to measure the complexity of the dynamics of heartbeat time series. Rényi entropy and weighted Rényi entropy are the entropy-like measures proposed in the multiscale analysis in which, with the new scheme, four parameters are involved. First, the influence of the variation of the new parameters in the entropy values is analyzed when different groups of subjects (with cardiac diseases or healthy) are considered. Secondly, we exploit the introduction of multiscale analysis in order to detect differences between the groups.

## 1. Introduction

Entropy-like measures are a powerful tool in the study of time series. The encoded information in a time series has turned out to be a valuable source of information. Since the seminal paper of Bandt and Pompe in 2002 [1], permutation entropy (PE) has been used to measure the complexity of different types of processes, since the order relation established in the time series allows finding patterns linking with the complexity of the system; see [2] for a review about permutation entropy and its applications. In particular, biomedical time series have been the focus of great interest, including neuronal signals and heart rate series [3]. Nevertheless, new variants of entropy-like measures have arisen in the last years, considering the nature of the series that is object of study [4], since differences appear when we consider different versions of entropy and different values of the parameters. Therefore, the analysis of the behavior of each of them, taking into account the final purpose that has been fixed, is relevant.

A natural approach to the study of possible cardiac problems is the analysis of heartbeat series. The activity of human beings influences heartbeat series, causing non-stationary series in healthy subjects. Cardiac diseases should be revealed in heartbeat series though significant changes in complexity. Difficulties in measuring that complexity have been considered and preliminary results can be found in different papers where the idea of extracting information from the time series is presented. Thus, Pincus and collaborators use approximate entropy (AE) in several papers to quantify the complexity and the regularity of biological time series [5,6]. In [7], symbolic dynamics and renormalized entropy were used to detect abnormalities in heart rate variability (HRV) in patients that had been classified as low-risk using traditional methods. In 2002, [8] a sample entropy analysis of neonatal heart rate variability was considered, taking into account the abnormal heart rate characteristics appearing in neonatal sepsis. Atrial fibrillation (AF) is a common cardiac arrhythmia in which irregular patterns of electrical activation are usually found in the atria [9]. Cammarota and Rogora (2005) studied the independence of non-stationary heartbeat series during atrial fibrillation (AF) comparing two methods, one using a linear Gaussian state space model and the other using symbolic permutation [10].

In [11], a complexity analysis of heart period variability under two different approaches was considered. The typification of complexity in short heart period variability series was done using Shannon entropy (SE) and conditional entropy (CE), the work concluded that the study of complexity can be very useful in the diagnosis of arrhythmias.

Entropy measures, approximate entropy (AE), sample entropy (SamE), fuzzy entropy (FE), and permutation entropy (PE) were computed for short EGG series in [12] in order to separate healthy patients and patients with congestive heart failure. In [13], Zunino and collaborators used permutation min-entropy (PME) in order to discriminate patients with AF. Recently, in 2018, permutation entropy and min-entropy in a heartbeat time series were applied to detect changes in the emotional states of subjects [14].

The quantification of the regularity in time series is the basic idea that underlies in this framework. The advantages of permutation entropy as a good measure of regular behavior and the simple, fast, and easy implementation and robustness and invariance with respect to non-linear monotonous transformations, see [1], makes it a valuable tool to analyze time series. Nevertheless, it is not clear what the best entropy-like measure to incorporate it is. The number of different entropy-like measures and the nature of the heartbeat series means that the election of an entropy-like measure good enough for measuring the complexity of the dynamics is non-trivial. Thus, Costa [15] highlighted that multiple time scales are inherent in healthy physiologic dynamics, and multiscale entropy can be adapted to study these types of processes. In particular, in [15] multiscale sample entropy is applied. On the other hand, other parameters should be taken into account to establish critical points in different groups of subjects for a more accurate discrimination. In fact, the delay parameter is in many situations fixed as τ=1 and is not included in the analysis, but this parameter can be non-trivial when we are considering heartbeat interval series, as has been highlighted in [13].

Our approach is aimed at analyzing different types of heartbeat series of 24 h for patients of three different groups (a congestive heart failure (CHF) group, healthy (H) group, and atrial fibrillation (AF) group) using multiscale analysis and discarding additional information with additional delay using Rényi permutation entropy (RPE), since this entropy-like measure has reported the best results in some biological processes [16], as well as weighted Rényi entropy. This means that two parameters are added to the original entropy-like measure that needs two parameters, namely, the embedding dimension *m* and the parameter associated with the Rényi parameter α, (recall that α<1 privileges rare events while α>1 does the same for frequent events). When the parameter α tends to 1, we obtain PE entropy. Summing up, four parameters are involved in multiscale Rényi permutation entropy (MRPE): the scale *s*, the delay τ, the embedding dimension *m*, and the Rényi parameter α. Analogously, weighted permutation entropy provides more control between the differences in the patterns, thus we include weighted multiscale Rényi entropy (WMRPE) parallel to (MRPE), as has been suggested in [17], where this scheme has been applied to the analysis of economic series; in particular, the analysis was focused on the closing prices of financial stock markets of different areas. Thus, we will simultaneously use two different scales in our analysis.

We consider the data freely available at http://www.physione.org/challenge/chaos, where a challenge entitled “Is the Normal Heart Rate Chaotic?” was launched and heart beat time series from 15 subjects are provided. Five of them correspond to patients suffering congestive heart failure (CHF), five come from subjects suffering atrial fibrillation (AF), and finally a group of five healthy subjects allows us to compare the results (control group). Our efforts are aimed at assessing if it is worth introducing multiscale analysis in heart time series. Hence, we will determine if any change and differences are detected when a multiscale analysis is added.

## 2. Terminology and Notation: Ordinal Patterns

Although most of the notions are well known, we recall them for the sake of completeness. The definitions that follow, with slightly differences, can be found in the literature; see for instance [1,3,4,18,19]. The starting point is a real time series ${\left(x_{n}\right)}_{n=1}^{T}$ where ordinal patterns of length *m* are considered. The length of the series must be big enough, since the procedure of reconstruction of the attractor evaluating the associated probability distribution requires enough data. Following [20], a required condition is T>5m!.

Let m∈N be a fixed natural number, $S_{m}$ be the group of permutations of length *m*, and $\pi =\left(r_{1},r_{2},\ldots ,r_{m}\right)\in S_{m}$. The vector $\left(x_{1},x_{2},\ldots ,x_{m}\right)\in R^{m}$ is said to be π-type if

$$x_{r_{1}}\leq x_{r_{2}}\leq \cdots x_{r_{m}}$$

and

$$r_{i-1}<r_{i},$$

if $x_{r_{i-1}}=x_{r_{i}}$ for i∈{2,…m}. Observe that the number of possible ordinal patterns of length *m* is given by the cardinality of $S_{m}$, that is $|S_{m}|=m!$.

The length of the patterns is called the embedding dimension. Following [1], low orders of *m* are recommended for practical purposes. We will use m=3,4, and 5.

The following definition, where delay parameter τ is included, gives the relative frequency of the pattern associated with the permutation $\pi \in S_{m}$.

**Definition** **1** **([1,4]).***Let ${\left(x_{n}\right)}_{n=1}^{T}$ be a time series, m∈N and $\pi \in S_{m}$, then the* relative frequency of *π, denoted by $p^{\tau }\left(\pi \right)$, is given by*

$$p^{\tau }\left(\pi \right)=\frac{\left|\{j:\left\{1,\ldots ,T-\left(m-1\right)\tau \right\}:\left(x_{j},x_{j+\tau },x_{j+2\tau },\ldots ,x_{j+(m-1)\tau }\right):\;is\;of\;\pi -type\}\right|}{T-(m-1)\tau }.$$

Permutation entropy (PE) was defined by Bandt and Pompe [1]. Let ${\left(x_{n}\right)}_{n=1}^{T}$ be a real time series, m,τ∈N. Then the permutation entropy (PE) is given by

$$\mathrm{PE}\left(m,\tau ,{\left(x_{n}\right)}_{n=1}^{T}\right)=-\sum p^{\tau }\left(\pi \right)\log\left(p^{\tau }\left(\pi \right)\right),$$

where $\pi \in S_{m}$ and τ∈N. The Rényi entropy variant [21] generalizes the permutation entropy [22]. The definition follows; see for instance [4] (Definition 4).

**Definition** **2.** *Let ${\left(x_{n}\right)}_{n=1}^{T}$ be a time series, α≠1, m,τ∈N. Then, the Rényi permutation entropy of ${\left(x_{n}\right)}_{n=1}^{T}$ (RPE), is given by*

$$\mathrm{RPE}\left(\alpha ,m,\tau ,{\left(x_{n}\right)}_{n=1}^{T}\right)=-\frac{1}{1-\alpha }\log\left(\sum_{\pi \in S_{m}}{\left(p^{\tau }\left(\pi \right)\right)}^{\alpha }\right),$$

*where $p^{\tau }\left(\pi \right)$ is given as in Definition 1. When the parameters are fixed, we write RPE for short.*

Permutation entropy (PE), also called Shannon entropy (SE), is obtained when α tends to 1 in Rényi permutation entropy. Liang [16] reported that the class of permutation entropy with the best results when EEG during different anesthesia states are considered is Rényi entropy. Moreover, the choice of the parameter is not a trivial question; see [23].

**Figure 1:**
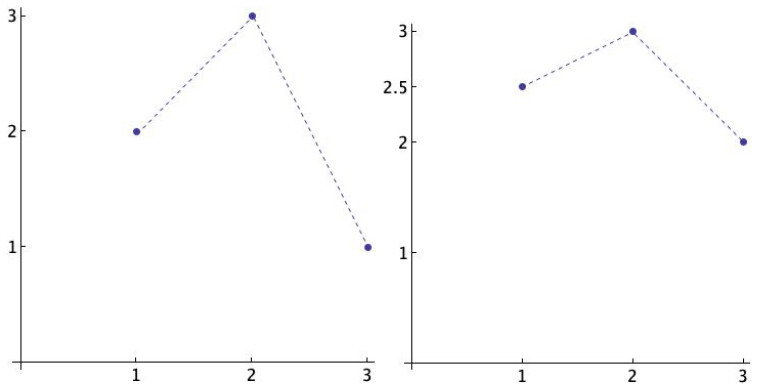
The ordinal pattern (3,1,2) is represented by two different vectors, (2,3,1) (left) and (2.5,3,2) (right). The distance between the values is clearly different.

The weighted version of Rényi entropy uses weighted frequencies in which not only the order is considered but also the differences between the values of the pattern. The idea is illustrated in Figure 1. Observe that (2,3,1) and (2.5,3,2) are the same pattern, namely (3,1,2), but the distance between the values are different. The definition follows.

**Definition** **3.** *Let ${\left(x_{n}\right)}_{n=1}^{T}$ be a time series, m∈N and $\pi \in S_{m}$, then the* weighted relative frequency of *π, denoted by $p_{\omega }^{\tau }\left(\pi \right)$, is given by*

$$p_{\omega }^{\tau }\left(\pi \right)=\frac{\sum_{j\in I(\pi )}\omega \left(j\right)}{\sum_{\pi \in S_{m}}\sum_{j\in I(\pi )}\omega \left(j\right)},$$

*where $I\left(\pi \right)=\{j\in \left\{1,\ldots ,T-\left(m-1\right)\tau \right\}:\;\left(x_{j},x_{j+\tau },x_{j+2\tau },\ldots ,x_{j+(m-1)\tau }\right):\;is\;of\;\pi -type\}$ for each $\pi \in S_{m}$, and*

$$\omega \left(j\right)=\frac{1}{m}\sum_{i=1}^{m}{\left(x_{j+(i-1)\tau }-\bar{x_{j}^{m,\tau }}\right)}^{2},$$

*and $\bar{x_{j}^{m,\tau }}=\frac{1}{m}\sum_{i=1}^{m}x_{j+(i-1)\tau }$.*

Now, weighted Rényi entropy (WRPE) is defined by

(1) $$\mathrm{WRPE}\left(\alpha ,m,\tau ,{\left(x_{n}\right)}_{n=1}^{T}\right)=-\frac{1}{1-\alpha }\log\left(\sum_{\pi \in S_{m}}{\left(p_{\omega }^{\tau }\left(\pi \right)\right)}^{\alpha }\right).$$

Normalized versions of entropy-like measures are obtained by dividing by log(m!), which is the maximal possible value for PE and RPE. We will consider the normalized versions throughout the paper.

The multiscale process that we will follow has different steps. The description follows. Let ${\left(x_{n}\right)}_{n=1}^{T}$ be a time series and s∈N the scale factor. The coarse-grained time series $\left(x_{n}^{s}\right)$ is given by

(2) $$x_{n}^{\left(s\right)}=\frac{1}{s}\sum_{i=(n-1)s+1}^{ns}x_{i},$$

for $1\leq n\leq [\frac{T}{s}]$, where $\left[\frac{T}{s}\right]$ denotes the integer part of $\frac{T}{s}$. We will consider for our analysis the range 1≤s≤20. Now, fixing the embedding dimension, *m*, the delay, τ, and the Rényi parameter, α, the multiscale Rényi permutation entropy is given by

(3) $$\mathrm{MRPE}\left(s,\alpha ,m,\tau ,{\left(x_{n}\right)}_{n=1}^{T}\right)=\mathrm{RPE}\left(\alpha ,m,\tau ,{\left(x_{n}^{\left(s\right)}\right)}_{n=1}^{\left[T/s\right]}\right).$$

whereas, the weighted multiscale Rényi permutation entropy (WMRPE) is given by

(4) $$\mathrm{WMRPE}\left(s,\alpha ,m,\tau ,{\left(x_{n}\right)}_{n=1}^{T}\right)=\mathrm{WRPE}\left(\alpha ,m,\tau ,{\left(x_{n}^{\left(s\right)}\right)}_{n=1}^{\left[T/s\right]}\right).$$

Observe that if s=1, then the MRPE (WMRPE) is simply the RPE (WRPE).

## 3. The Data

**Figure 2:**
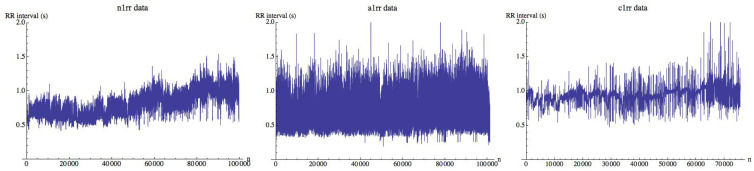
Heart beat interval series n1 (**left**), a1 (**middle**), and c1 (**right**). In each figure, heart beat (RR) intervals (in seconds) are plotted.

The data corresponds to a collection of 15 heart beat intervals (RR-interval) freely available on Physionet (http://www.physionet.org/challenge/chaos). Time series n1rr, n2rr, n3rr, n4rr, and n5rr correspond to healthy subjects, a1rr, a2rr, a3rr, a4rr, and a5rr are time series in atrial fibrillation, and finally c1rr, c2rr, c3rr, c4rr, and c5rr are time series in congestive heart failure. Time series are not filtered and were obtained from continuous ambulatory (Holter) electrocardiograms. Each time series is about 24 h long (roughly 100,000 intervals). More information about the recordings referring to healthy and congestive heart failure subjects can be obtained on the web. No additional details about the time series in atrial fibrillation are given. Figure 2 shows the data for a subject in each group. Although they are different, in some cases is not easy to classify a subject only by visual inspection. One anonymous reviewer has suggested the possibility of analyzing the differences between the groups when the images are similar using a fuzzy technique of quantification of the distances in digital images using fuzzy divergence [24], which is quite a different approach from this one.

## 4. Numerical Analysis

**Figure 3:**
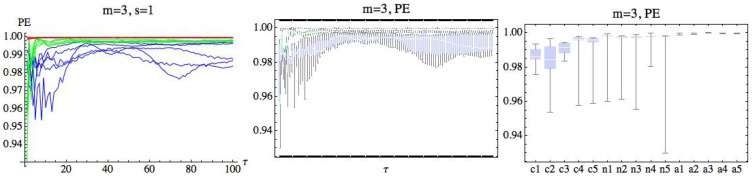
In the left, the permutation entropy (PE) for m=3 and s=1 is represented for each subject. The blue color represents the congestive heart failure (CHF) group, green has been used for the healthy (H) group, and red for the atrial fibrillation (AF) group. The delay parameter τ has been represented in the OX axis. In the middle, the same situation has been represented using box plots, grouping the subjects belonging to the same group. Finally, in the right, we have taken the mean with respect to τ when 1≤τ≤100 for each subject and have represented the corresponding box plot.

A preliminary numerical approach computing PE gives us the general idea that entropy values from a subject in the AF group are higher than the other groups (see Figure 3), where (PE) has been computed for each subject, taking m=3 and s=1. The delay parameter τ∈N takes values in the range 1≤τ≤100. The CHF group has been drawn in blue, green has been used for the H group, and the values obtained from the AF group have been represented in red. The deviation of the values of PE entropy with respect to its mean for each subject in the AF group is smaller than the rest of the groups. A simple look at the graphics reveals differences between the groups. We can also observe that entropy values that come from the CHF group and the healthy group are mixed and are not easy to distinguish.

**Figure 4:**
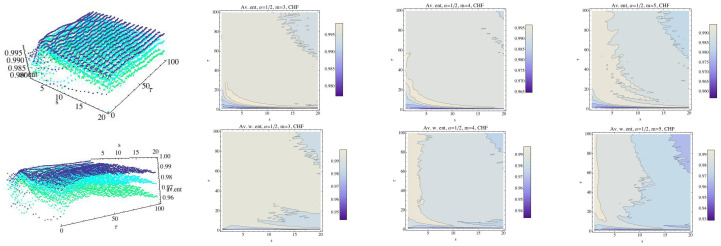
Average of Rényi entropy and average weighted Rényi entropy for the CHF group and $\alpha =\frac{1}{2}$. We have fixed m=3 (dark blue), m=4 (light blue), and m=5 (green). The top row is for the Rényi permutation entropy (RPE) and the bottom one for the weighted Rényi entropy (WRPE). The projection of the values (contour plot) of the entropy for each fixed embedding dimension for the CHF group have been drawn in 2D graphs.

**Figure 5:**
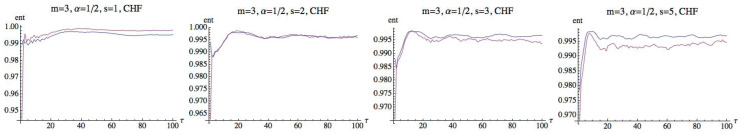
Average Rényi entropy (RPE) (blue) and average weighted Rényi entropy (WRPE) (red) for the CHF group with $\alpha =\frac{1}{2}$, m=3, and s=1,2,3, and 5. Observe that for s=1, the WRPE is higher than the RPE for most values of τ. Nevertheless, for s≥3 the situation changes.

**Figure 6:**
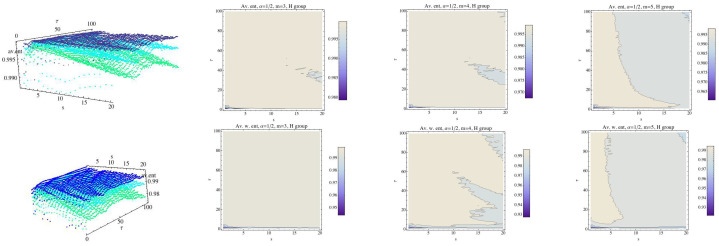
Average Rényi entropy in the top row and average weighted Rényi entropy in the bottom row for the H group and $\alpha =\frac{1}{2}$. We have fixed m=3 (dark blue), m=4 (light blue), and m=5 (green). The projections of the entropy for each fixed embedding dimension for the H group have been drawn in 2D graphs.

**Figure 7:**
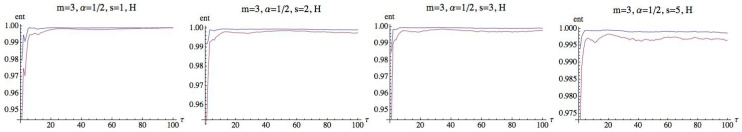
Average Rényi entropy (blue) and average weighted Rényi entropy (red) for the H group and $\alpha =\frac{1}{2}$, m=3, and s=1,2,3, and 5.

**Figure 8:**
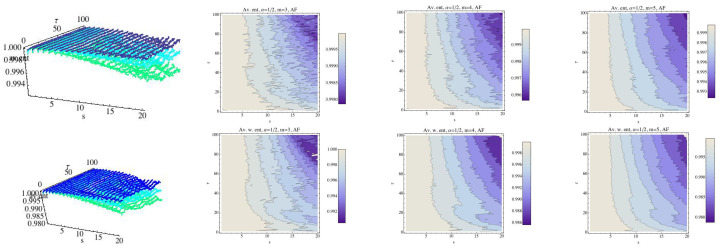
Average Rényi entropy in the top row and average weighted Rényi entropy in the bottom row for the AF group and $\alpha =\frac{1}{2}$. We have fixed m=3 (dark blue), m=4 (light blue), and m=5 (green). The contour of the entropy for each fixed embedding dimension for the AF group has been drawn In 2D graphs.

**Figure 9:**
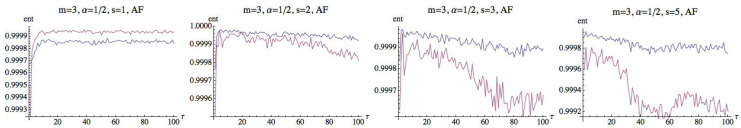
Average Rényi entropy (RPE) (blue) and average weighted Rényi entropy (WRPE) (red) for the AF group and $\alpha =\frac{1}{2}$, m=3, and s=1,2,3, and 5.

**Figure 10:**
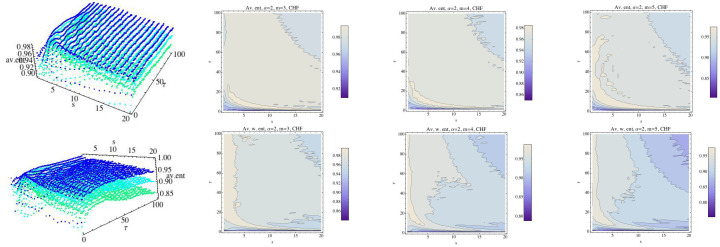
Average Rényi entropy in the top row and average weighted Rényi entropy in the bottom row for the CHF group and α=2. We have fixed m=3 (dark blue), m=4 (light blue), and m=5 (green). The projections of the entropy for each fixed embedding dimension for the CHF group have been drawn in 2D graphs.

**Figure 11:**
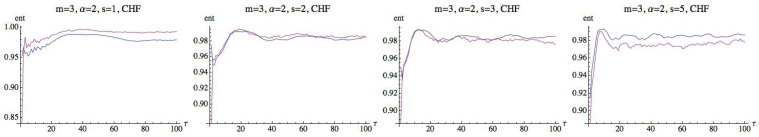
Average Rényi entropy (RPE, blue) and average weighted Rényi entropy (WRPE, red) for the CHF group and α=2, m=3, and s=1,2,3, and 5. Observe that for s=1, the WRPE is higher for most values of τ; nevertheless, for s=5 the situation changes.

**Figure 12:**
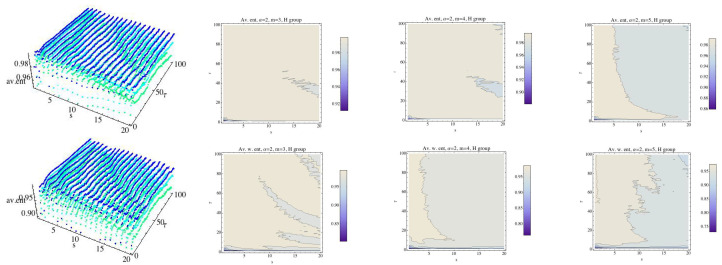
Average Rényi entropy and average weighted Rényi entropy for the H group and α=2. We have fixed m=3 (dark blue), m=4 (light blue), and m=5 (green). The projections of the entropy for each fixed embedding dimension for the H group have been drawn in 2D graphs.

**Figure 13:**
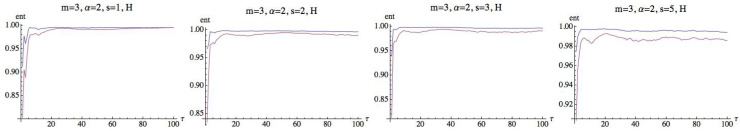
Average Rényi entropy (RPE, blue) and average weighted Rényi entropy (WRPE, red) for the H group and α=2, m=3, and s=1,2,3, and 5.

**Figure 14:**
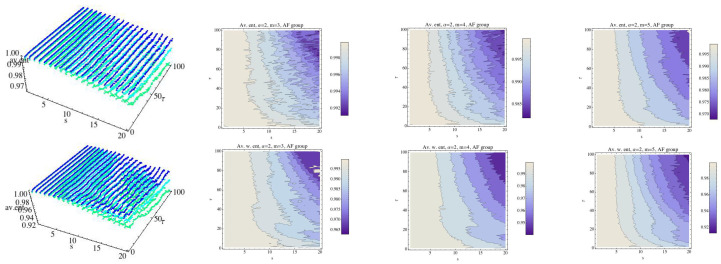
Average Rényi entropy in the top row and average weighted Rényi entropy in the bottom row for the AF group and α=2. We have fixed m=3 (dark blue), m=4 (light blue), and m=5 (green). The contour plots of the entropy for each fixed embedding dimension for the AF group have been drawn in 2D graphs.

In [13] permutation min-entropy is used to analyze this data collection. Here, we will analyze these series using normalized MRPE and WMRPE entropies. All procedures have been implemented with a code “ad hoc”. First, we analyze the behavior of each group (the mean) for the embedding dimension m=3, m=4, and m=5, taking into account that when the value of *m* increases, so does the computational time. We fix $\alpha =\frac{1}{2}$ and α=2 as reference values. Regarding the scale factor *s*, it ranges over the interval 1≤s≤20 and τ ranges over 1≤τ≤100. The results are shown in Figure 4, Figure 5, Figure 6, Figure 7, Figure 8, Figure 9, Figure 10, Figure 11, Figure 12, Figure 13 and Figure 14. In Figure 4, we have fixed $\alpha =\frac{1}{2}$ and have computed the average of RPE and WRPE for the CHF group, considering the embedding dimensions m=3 (dark blue), m=4 (light blue), and m=5 (green) in the 3D graph. We can observe in the contour plots that changes in the values of the entropy, that is, changes in the complexity of the dynamics, are presented for values close to the boundary, namely τ=1 and s=1. When the value of the embedding dimension increases, so does the rate at which the entropy decreases with respect to parameter values τ and *s*. We can see that the variability of the values of the entropy for $\alpha =\frac{1}{2}$ when τ and *s* increase is greater when *m* grows, although the values of the entropy are lower. Finally, for a fixed τ, changes in the values of the scale parameter *s* means changes in the values of the RPE and in the values of the WRPE.

Regarding the relationship between the WRPE and the RPE for a fixed *m* and $\alpha =\frac{1}{2}$, we can observe that it depends on the scale value *s*; see Figure 5, where the RPE is drawn in blue and the WRPE in red. This relationship can remain unnoticed if multiscale analysis is not involved. Thus, for s=1 we can observe that for τ≥3, the WRPE is greater than or equal to the RPE. Nevertheless, when s=2 the order relation changes and there is no rule to determine if an entropy measure is greater than the other one. For values of s≥3, the relationship turns around, and the RPE is greater than the WRPE. The limit case τ=1 and close values of τ to this gives a different behavior.

Fixing $\alpha =\frac{1}{2}$, we compute the average of the RPE and WRPE for the H group. We have drawn RPE in the top row for m=3 (dark blue), m=4 (light blue), and m=5 (green) and the contour plots for each value of the embedding dimension *m*; see Figure 6. The bottom row shows the results obtained for the WRPE. Again, roughly speaking, as *m* increases the values of Rényi entropy decreases. We can see that the behavior with respect to the projections for the CHF and H groups are different. Moreover, in the case of the H group, the values of the weighted Rényi entropy are lower than those of the Rényi entropy for most values of τ, even for the case of s=1, compared to what happened in the case of CHF group; see Figure 7. We recall that the CHF group and the H group were not easy to distinguish. and all the differences must be taken into account.

Finally, we compute the RPE and WRPE for the AF group following the previous schedule. The results are shown in Figure 8, and they are quite different from the results that were obtained for the previous groups. The relationship between the values of RPE and WRPE for m=3 are shown in Figure 9. Again, for s=1 we can see that there are values of τ for which the WRPE is greater than the RPE, and the situation changes when *s* increases. Thus, only the H group has a different behavior in respect to this fact.

**Figure 15:**
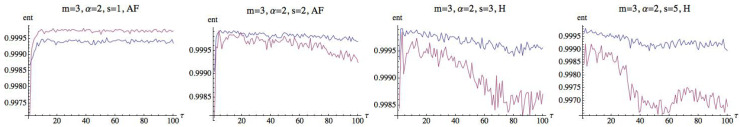
Average of Rényi entropy (RPE, blue) and average of weighted Rényi entropy (WRPE, red) for the AF group and $\alpha =\frac{1}{2}$, m=3, and s=1,2,3, and 5.

The above computations are now made for α=2, and we observe the behavior of each group for this value, which is greater than 1. Results are shown in Figure 10, Figure 11, Figure 12, Figure 13, Figure 14 and Figure 15.

For s=1 and s=2 there are values of τ such that the WRPE is greater than the RPE. Figure 11 shows the RPE (blue) and WRPE (red) results for m=3 and s=1, s=2, s=3, and s=5.

Following the previous analysis, we have been able to observe some differences between them. Now, we are interested in comparing the groups by analyzing what are the best parameter values to differentiate the three groups. For that, we compute the entropies of each subject and take the maximum and the minimum value in each group for each fixed parameter value. More concretely, fixing the parameter values *s*, *m*, α, and τ, we say that two groups are differentiated by the entropy measure if the intervals limited by their corresponding maximum and minimum values are disjoint, and the difference between two groups is the minimum distance between any two values belonging to different groups. Namely, fixing *m*, α, *s*, and τ, we compute for a group *G*

$$M_{G}\left(s,\alpha ,m,\tau \right)=\max\left\{\mathrm{MRPE}\left(s,\alpha ,m,\tau ,{\left(x_{n}\right)}_{n=1}^{T}\right):\;{\left(x_{n}\right)}_{n=1}^{T}\in G\right\},$$

$$m_{G}\left(s,\alpha ,m,\tau \right)=\min\left\{\mathrm{MRPE}\left(s,\alpha ,m,\tau ,{\left(x_{n}\right)}_{n=1}^{T}\right):\;{\left(x_{n}\right)}_{n=1}^{T}\in G\right\},$$

$$M_{G}^{w}\left(s,\alpha ,m,\tau \right)=\max\left\{\mathrm{WMRPE}\left(s,\alpha ,m,\tau ,{\left(x_{n}\right)}_{n=1}^{T}\right):\;{\left(x_{n}\right)}_{n=1}^{T}\in G\right\},$$

$$m_{G}^{w}\left(s,\alpha ,m,\tau \right)=\min\left\{\mathrm{WMRPE}\left(s,\alpha ,m,\tau ,{\left(x_{n}\right)}_{n=1}^{T}\right):\;{\left(x_{n}\right)}_{n=1}^{T}\in G\right\}.$$

Fixing *s*, α, *m*, and τ, we say that group $G_{1}$ and group $G_{2}$ are differentiated by the MRPE (resp. WMRPE) if

$$\left[m_{G_{1}}\left(s,\alpha ,m,\tau \right),M_{G_{1}}\left(s,\alpha ,m,\tau \right)\right]\cap \left[m_{G_{2}}\left(s,\alpha ,m,\tau \right),M_{G_{2}}\left(s,\alpha ,m,\tau \right)\right]=\emptyset ,$$

(resp. $\left[m_{G_{1}}^{w}\left(s,\alpha ,m,\tau \right),M_{G_{1}}^{w}\left(s,\alpha ,m,\tau \right)\right]\cap \left[m_{G_{2}}^{w}\left(s,\alpha ,m,\tau \right),M_{G_{2}}^{w}\left(s,\alpha ,m,\tau \right)\right]=\emptyset$).

**Figure 16:**
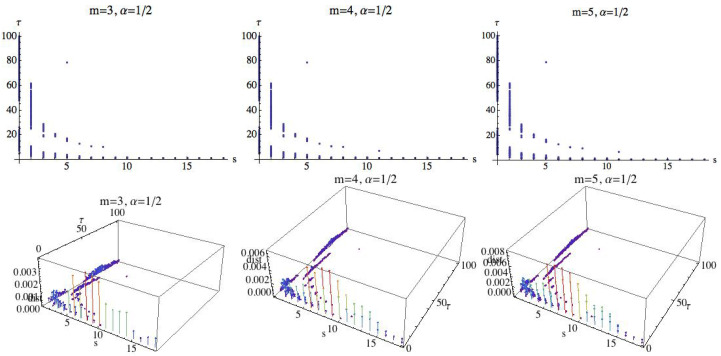
For $\alpha =\frac{1}{2}$, values of *s* and τ for which the three groups are separated by the multiscale Rényi permutation entropy (MRPE). In the 3D graphs, the OX axis represents the parameter *s*, where 1≤s≤20, in the OY axis is represented the parameter τ, 1≤τ≤100, and finally in the OZ axis is represented the difference between the three groups, when the groups are differentiated by the MRPE.

We will investigate the existence of parameter values in which the three groups are differentiated simultaneously. It means that a relationship of order G1>G2>G3 should be given. In addition, in those situations we will compute the difference between G1 and G2 and the difference between G2 and G3, and we will compute the minimum of both. This value will be called the difference between the three groups. We are interested in finding the parameter values by which the groups are differentiated and the difference is maximal. Observe that although the number of subjects is small, a negative answer to this question also gives valuable information. For this, we fix α and consider three different cases: m=3, m=4, and m=5. Figure 16 shows the results for $\alpha =\frac{1}{2}$. In 3D graphs (Figure 16), the OX axis represents the parameter *s*, where 1≤s≤20, in the OY axis is represented the parameter τ, 1≤τ≤100, and finally in the OZ axis is represented the difference between the three groups, when the groups are differentiated by MRPE. The 2D graphs show the values (s,τ) for which the three groups are disjoint in the sense described above. Whereas in the 3D plots we can see not only the parameter values in which the three groups are disjoint but also the difference between them. When *m* changes, the shape of the distribution of the points (s,τ) is similar although the number of points increases, namely for m=3 there are 168 points, for m=4 there are 283 points, and for m=5 there are 348 points. The maximum difference increases with the parameter *m*, as we can see in Figure 16. The difference attains its maximum values when τ=1. The relation of order between the three groups is, in all cases, AF > H > CHF. More details are given in Table 1, where the results for m=3 and $\alpha =\frac{1}{2}$ are shown.

When we compute the WMRPE instead of the MRPE, the results are different. The values that appear in Table 2 show that in general terms, the WMRPE is worse than the MRPE for differentiating the three groups.

**Figure 17:**
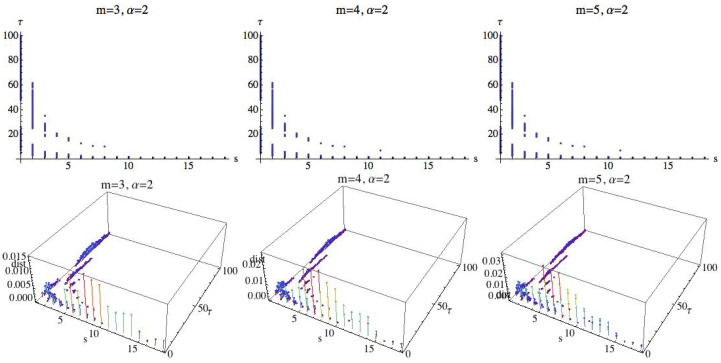
Fixing α=2, values of *s* and τ for which the three groups are separated by the MRPE are shown. In the 3D graphs, the OX axis represents the parameter *s*, where 1≤s≤20, in the OY axis is represented the parameter τ, 1≤τ≤100, and finally in the OZ axis is represented the difference between the three groups, when the groups are differentiated by the MRPE.

Now we consider a fixed value of α greater than 1; we follow with the reference value α=2. Figure 17 shows the results. The shape and the distribution of the points (s,τ) for a fixed embedding dimension is similar to the results obtained for $\alpha =\frac{1}{2}$, but we can observe that the maximum difference is greater than the one obtained in the above case.

For α=2, when the weighted version is considered, we obtain the points and the differences that have been collected in Table 3, taking 3≤m≤5, 1≤s≤20, and 1≤τ≤100.

**Figure 18:**
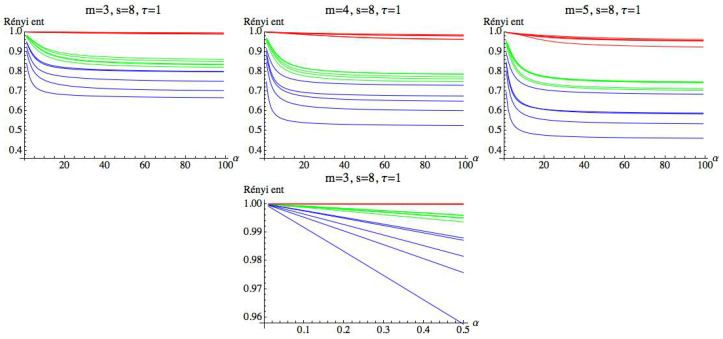
The top row shows the RPE for s=8, τ=1 and m=3 (left), m=4 (middle), and m=5 (right). In red is the AF group, in green is the H group, and the CHF results are in blue. The OX axis represents α∈[2,100]. and the RPE is represented in the OY axis. The bottom graph shows the results for m=3, s=8, and τ=1 for α∈(0,0.5).

Following the previous results, we see that the maximum value has been attained for s=8 and τ=1. To see the influence of the parameter α, we set s=8 and τ=1, and we compute the RPE values in the OY axis for each value of α (represented in the OX axis); see Figure 18. We can see that when α increases, the entropy values tend to a limit value.

**Figure 19:**
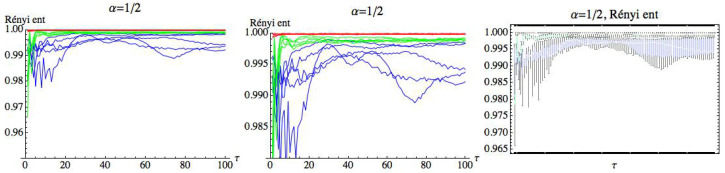
Rényi entropy for $\alpha =\frac{1}{2}$ and embedding dimension m=3. We have drawn the Rényi entropy for the CHF group in blue, for the healthy group in green, and for the AF group in red, on the left. A zoomed-in view of the right figure is shown in the middle, where the differences between the three groups when τ runs over the range can be appreciated. Finally, box plots for each group have been represented on the right.

**Figure 20:**
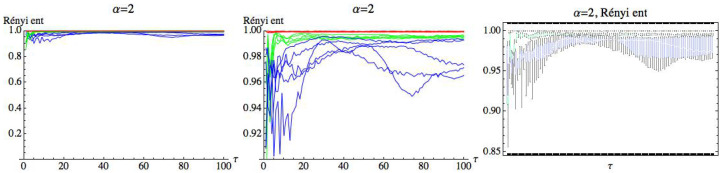
Rényi entropy for α=2 and embedding dimension m=3. We have drawn the Rényi entropy for the CHF group in blue, for the healthy group in green, and for the AF group in red on the left.

**Figure 21:**
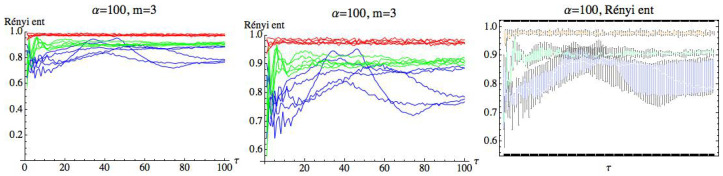
Rényi entropy for α=100 and embedding dimension m=3. We have drawn the Rényi entropy for the CHF group in blue, for the healthy group in green, and for the AF group in red on the left. A zoomed-in view of the right figure is shown in the middle, where the differences between the three groups when τ runs over the range can be appreciated. Finally, box plots for each group have been represented on the right.

For s=1, we can observe that there is a large quantity of values of τ for which the three groups are differentiated, although the differences in these values are significantly lower than in the previous case. For s=1, we simply get RPE with delay, τ, that now runs over a set of cardinality equal to 100. This means that for a fixed *m*, we have two parameters α and τ. Fixed the embedding dimension m=3, we compute the Rényi entropy for τ∈N taking values in the interval 1≤τ≤100. Figure 19, Figure 20 and Figure 21 show the results for $\alpha =\frac{1}{2}$, α=2, and α=100, respectively. We can see that the AF group has a different behavior from the healthy and CHF groups. It is possible to find values of τ for which the three groups can be differentiated. More details are included in the Appendix A (Table A1 and Table A2), where different values of α have been considered for m=3 and s=1. It is observed that when α decreases, the entropy values of the different groups are closer.

## 5. Conclusions

In this paper, we propose a multiscale analysis with RPE and WRPE to analyze heartbeat time series, where four parameters are considered in the study of the behavior of a heartbeat time series. Our purposes are twofold. On the one hand, we have considered the behavior of the entropy measure when parameters change in a range. The introduction of new parameters makes the analysis more difficult and more expensive in computational terms, but on the other hand, when we add new parameters, this allows a better fit to the research objective. From this point of view, it is natural to ask if the introduction of two additional scales gives non-redundant information. In this direction, numerical analysis shows differences between the three groups considered, namely a congestive heart failure (CHF) group, healthy (H) group, and atrial fibrillation (AF) group. Moreover, an additional characteristic for the healthy group is obtained, as we can see that this group has a different behavior in the relationship between the RPE and WRPE when *s* changes. On the other hand, we compare the results obtained for the RPE and WRPE, and we find the values for which the three groups can be differentiated using segments limited by the minimum and maximum values that have been attained in each group. The groups are called differentiated if the intervals are disjoint. In addition, we compute the values of *s* and τ that simultaneously differentiate the three groups, taking the two reference values of $\alpha =\frac{1}{2}$ and α=2 and embedding dimensions m=3,4 and 5. The numerical approach shows that even the RPE is better to differentiate than the WRPE.

The results look promising in the sense that they show that the introduction of multiscale analysis of Rényi entropy reveals more details about the behavior of each group and highlights non-redundant differences between them. The results that have been obtained are a first step in the idea that multiscale analysis should be taken into account to obtain an adequate interpretation of ordinal patterns in physiological terms, in order to consider all of the potential information that they can reveal. 

## Figures and Tables

**Table 1:** For m=3 and $\alpha =\frac{1}{2}$, values of *s* and τ for which the three groups are disjoint.

*s*	τ
1	5, 7, 8, 9, 10, 11, 12, 13, 14, 15, 16, 17, 18, 19, 20, 21, 22, 23, 24, 26, 48, 49, 50, 51, 52, 53, 54, 55, 56, 57, 58, 59, 60, 61, 62, 63, 64, 65, 66, 67, 68, 69, 70, 71, 72, 73, 74, 75, 76, 77, 78, 79, 80, 81, 82, 83, 84, 85, 86, 87, 88, 89, 90, 91, 92, 93, 94, 95, 96, 97, 98, 99, 100
2	3, 4, 5, 6, 7, 8, 9, 10, 11, 25, 26, 28, 29, 30, 31, 32, 33, 34, 35, 36, 37, 38, 39, 40, 41, 42, 43, 44, 45, 46, 47, 48, 49, 50, 51, 52, 53, 54, 55, 56, 58, 59, 60, 61, 62
3	2, 3, 4, 5, 6, 19, 20, 23, 24, 25, 26, 27, 28, 29
4	2, 3, 4, 5, 19, 20, 21
5	2, 3, 4, 15, 16, 17, 79
6	1, 2, 3, 13
7	1, 2, 11
8	1, 2, 10
9	1, 2
10	1, 2
11	1
12	1
13	1
14	1
15	1
16	1
17	1
18	1

**Table 2:** Fixing $\alpha =\frac{1}{2}$, values for the parameters m,s,τ∈N in the ranges 3≤m≤5, 1≤s≤20, and 1≤τ≤100 such that the CHF group, H group, and AF group are disjoint. The difference is included, as well as the order relation between them.

*m*	*s*	τ	Difference	Relation
3	1	3	0.000111612	CHF>AF>H
4	1	3	0.000111612	CHF>AF>H
5	1	3	0.000111612	CHF>AF>H
5	11	1	0.0018635	AF>H>CHF
5	6	2	0.00249138	AF>H>CHF

**Table 3:** Fixing α=2, values for the parameters m,s,τ∈N in the ranges 3≤m≤5, 1≤s≤20, and 1≤τ≤100 such that the CHF group, H group, and AF group are disjoint. The difference and the order relation between them are also included.

*m*	*s*	τ	Difference	Relation
3	1	3	0.000435339	CHF>AF>H
4	11	1	0.00213423	AF>H>CHF
4	6	2	0.00461844	AF>H>CHF
4	1	3	0.000435339	CHF>AF>H
5	11	1	0.00213423	AF>H>CHF
5	6	2	0.00461844	AF>H>CHF
5	9	1	0.0105166	AF>H>CHF
5	10	1	0.00585264	AF>H>CHF
5	5	2	0.00935592	AF>H>CHF
5	1	3	0.000435339	CHF>AF>H

## Appendix A

**Table A1:** PE for m=3 and 1≤τ≤100.

	c1rr		c2rr		c3rr		c4rr		c5rr	
*s*	Mean	σ	Mean	σ	Mean	σ	Mean	σ	Mean	σ
1	0.565761	0.0266769	0.559309	0.0290653	0.567659	0.0230525	0.544987	0.0357687	0.540677	0.0379
25	0.565925	0.0263007	0.559512	0.0288102	0.567692	0.0233685	0.54487	0.0355832	0.540604	0.0382577
50	0.56497	0.0270585	0.559621	0.0286887	0.567367	0.023753	0.544424	0.0354137	0.544424	0.0354137
75	0.56529	0.0264484	0.559033	0.0284339	0.567506	0.0236121	0.544782	0.0354761	0.53852	0.0398734
100	0.564318	0.0265197	0.559584	0.029156	0.567742	0.02381	0.543665	0.036225	0.541077	0.0363404
	n1rr		n2rr		n3rr		n4rr		n5rr	
*s*	Mean	σ	Mean	σ	Mean	σ	Mean	σ	Mean	σ
1	0.543486	0.036892	0.544803	0.0366675	0.54408	0.0362984	0.554415	0.0322511	0.528802	0.040533
25	0.543122	0.0371364	0.54011	0.037032	0.545062	0.036156	0.554613	0.01318338	0.528907	0.040142
50	0.543282	0.0373946	0.54417	0.037435	0.544899	0.0359118	0.554543	0.0317886	0.52937	0.0405128
75	0.543006	0.038461	0.54417	0.0382501	0.545281	0.035161	0.554957	0.0314647	0.52946	0.0405214
100	0.543139	0.0380009	0.542742	0.0385849	0.544892	0.0355305	0.553622	0.0321451	0.528615	0.040817
	a1rr		a2rr		a3rr		a4rr		a5rr	
*s*	Mean	σ	Mean	σ	Mean	σ	Mean	σ	Mean	σ
1	0.573109	0.0199455	0.574673	0.0185836	0.575351	0.0180058	0.574673	0.01885836	0.574673	0.0185836
25	0.573017	0.0196502	0.574934	0.0181619	0.575338	0.0179431	0.574934	0.0181619	0.574934	0.0181619
50	0.573126	0.0196991	0.575128	0.0174678	0.575286	0.0178132	0.575128	0.0174678	0.575128	0.0174678
75	0.572824	0.0199265	0.574989	0.0190529	0.575573	0.0172598	0.574989	0.0190529	0.574989	0.0190529
100	0.573328	0.0188351	0.574735	0.0175133	0.575098	0.0183671	0.574735	0.0175133	0.574735	0.0175133

**Table A2:** Mean and standard deviation for Rényi entropy taking m=3 and 1≤τ≤100 for different values of α.

	**Rényi Entropy**m=3, 1≤τ≤100									
	$\alpha =\frac{1}{2}$		$\alpha =\frac{1}{3}$		$\alpha =\frac{1}{5}$		$\alpha =\frac{1}{10}$		$\alpha =\frac{1}{100}$	
	Mean	σ	Mean	σ	Mean	σ	Mean	σ	Mean	σ
c1	0.99375	0.0016742	0.995901	0.00108809	0.997573	0.000639407	0.998799	0.000314675	0.999881	0.0000310164
c2	0.992373	0.00440852	0.994975	0.00288105	0.997013	0.00170015	0.998518	0.00083924	0.999853	0.0000829394
c3	0.995505	0.00135784	0.997034	0.000876451	0.998235	0.000512168	0.999123	0.000250985	0.999913	0.0000246431
c4	0.997599	0.00231847	0.998417	0.00151459	0.999059	0.000893696	0.999533	0.000441177	0.999954	0.0000436064
c5	0.996863	0.00300583	0.997932	0.00197033	0.998771	0.00116577	0.99939	0.000576648	0.999939	0.0000570993
n1	0.998866	0.00217053	0.999249	0.00143686	0.999552	0.000856727	0.999777	0.000426199	0.999978	0.0000424152
n2	0.998122	0.00255504	0.99876	0.00168197	0.999262	0.000998495	0.999633	0.000495129	0.999963	0.0000491351
n3	0.99842	0.00220677	0.998955	0.00145612	0.999377	0.000865973	0.99969	0.000429973	0.999969	0.0000427177
n4	0.99882	0.00108625	0.999219	0.000717013	0.999535	0.000426684	0.999768	0.000211997	0.999977	0.000021077
n5	0.998207	0.00337506	0.998817	0.00221474	0.999296	0.00131093	0.99965	0.000648507	0.999965	0.0000642098
a1	0.999883	0.0000746988	0.999922	0.0000496687	0.999953	0.0000297382	0.999977	0.0000148454	0.999998	$1.48239*10^{-6}$
a2	0.99982	0.0000692071	0.99988	0.0000460782	0.999928	0.0000276183	0.999964	0.0000137984	0.999996	$1.37888*10^{-6}$
a3	0.999918	0.0000260503	0.999945	0.0000173427	0.999967	0.0000103941	0.999984	$5.19273\times 10^{-6}$	0.999998	$5.18886\times 10^{-7}$
a4	0.999821	0.0000319789	0.999881	0.0000213546	0.999929	0.0000128298	0.999964	$6.42139\times 10^{-6}$	0.999996	$6.42724\times 10^{-7}$
a5	0.999809	0.0000419525	0.999873	0.0000279582	0.999924	0.0000167702	0.999962	$8.38334\times 10^{-6}$	0.999996	$8.38178\times 10^{-7}$
	α=2		α=3		α=5		α=10		α=100	
	Mean	σ	Mean	σ	Mean	σ	Mean	σ	Mean	σ
c1	0.971488	0.00810825	0.954373	0.0131568	0.919262	0.022572	0.857684	0.0325554	0.782297	0.0321941
c2	0.96679	0.0200377	0.948698	0.0309635	0.915286	0.049456	0.864263	0.072046	0.800394	0.0849372
c3	0.980388	0.00696758	0.969188	0.0117617	0.945976	0.0220604	0.90024	0.0384958	0.82708	0.0428283
c4	0.989489	0.0106662	0.983489	0.0166264	0.970898	0.0267743	0.941862	0.0390777	0.871617	0.0415113
c5	0.986294	0.0134194	0.978577	0.0205494	0.962802	0.0320426	0.928468	0.0437329	0.85563	0.0435773
n1	0.995255	0.00885824	0.992754	0.0129209	0.987708	0.0191147	0.975157	0.0261347	0.918917	0.0280182
n2	0.991919	0.0109831	0.987451	0.0164899	0.978208	0.0251411	0.955622	0.0341087	0.887612	0.0347691
n3	0.993263	0.00924895	0.989563	0.0136436	0.981805	0.0203264	0.961995	0.0276005	0.896366	0.0297364
n4	0.994969	0.00465057	0.992169	0.00711745	0.98615	0.011609	0.970086	0.0179958	0.907732	0.0205302
n5	0.992308	0.0144336	0.988136	0.0209432	0.979637	0.0296429	0.958767	0.0378302	0.892409	0.0385716
a1	0.999529	0.00030556	0.99929	0.000464605	0.998802	0.000792727	0.997539	0.00164017	0.980157	0.00615212
a2	0.999274	0.000280116	0.998906	0.000423513	0.998161	0.000716948	0.996254	0.00148206	0.974081	0.00573953
a3	0.999669	0.000105529	0.9995	0.000159653	0.999158	0.00027073	0.998272	0.00056533	0.984414	0.00439242
a4	0.999277	0.000126117	0.998908	0.000187545	0.998155	0.000307843	0.996192	0.000600026	0.972335	0.00283459
a5	0.999225	0.000168423	0.998827	0.000253341	0.998015	0.000424909	0.995882	0.000865272	0.970802	0.00409266
